# TrmBL2 from *Pyrococcus furiosus* Interacts Both with Double-Stranded and Single-Stranded DNA

**DOI:** 10.1371/journal.pone.0156098

**Published:** 2016-05-23

**Authors:** Sebastian Wierer, Peter Daldrop, Misbha Ud Din Ahmad, Winfried Boos, Malte Drescher, Wolfram Welte, Ralf Seidel

**Affiliations:** 1 Department of Chemistry, University of Konstanz, Universitätsstraße 10, 78464, Konstanz, Germany; 2 Department of Biology, University of Konstanz, Universitätsstraße 10, 78464, Konstanz, Germany; 3 Institute for Molecular Cell Biology, University of Münster, Schlossplatz 5, 48149, Münster, Germany; 4 Institute of Experimental Physics I, Universität Leipzig, Linnéstr. 5, 04103, Leipzig, Germany; Keio University, JAPAN

## Abstract

In many hyperthermophilic archaea the DNA binding protein TrmBL2 or one of its homologues is abundantly expressed. TrmBL2 is thought to play a significant role in modulating the chromatin architecture in combination with the archaeal histone proteins and Alba. However, its precise physiological role is poorly understood. It has been previously shown that upon binding TrmBL2 covers double-stranded DNA, which leads to the formation of a thick and fibrous filament. Here we investigated the filament formation process as well as the stabilization of DNA by TrmBL2 from *Pyroccocus furiosus* in detail. We used magnetic tweezers that allow to monitor changes of the DNA mechanical properties upon TrmBL2 binding on the single-molecule level. Extended filaments formed in a cooperative manner and were considerably stiffer than bare double-stranded DNA. Unlike Alba, TrmBL2 did not form DNA cross-bridges. The protein was found to bind double- and single-stranded DNA with similar affinities. In mechanical disruption experiments of DNA hairpins this led to stabilization of both, the double- (before disruption) and the single-stranded (after disruption) DNA forms. Combined, these findings suggest that the biological function of TrmBL2 is not limited to modulating genome architecture and acting as a global repressor but that the protein acts additionally as a stabilizer of DNA secondary structure.

## Introduction

The archaeal “TrmB-like” protein TrmBL2 (30.6 kDa) was discovered during a search for homologues of the newly discovered protein TrmB (38 kDa) [1]. This name-giving member of the family functions in the hyperthermophilic archaea *Thermococcus litoralis* and *Pyrococcus furiosus* as a transcriptional repressor of the TM genes, which encode the ABC transporter for trehalose and maltose [2]. It also controls the corresponding MD genes for maltotriose and sucrose transport [3]. TrmB family proteins are mainly found in euryarchaeal members of the order *Thermococcales* containing the genera *Pyrococcus*, *Thermococcus* and *Paleococcus* [4] with optimal growth temperatures between 80°C and 100°C. TrmBL1, 2, 3 and TrmBL4 from this family share significant sequence similarity with TrmB within the N-terminal 120 residues comprising the DNA binding ewHTH (extended winged helix-turn-helix) domain. TrmBL1 has been found to act as both, activator for certain gluconeogenic genes and repressor for glycolytic enzymes, possibly acting as a switch between different metabolic states [5,6]. Another TrmB-like protein, VNG1451C from the halophilic archaeon *Halobacterium salinarum* NRC-1, controls genes encoding glycolytic and gluconeogenic pathways, purine biosynthesis, cobalamin biosynthesis, the tricarboxylic acid cycle, and glutamate synthesis [7]. Sequence similarities [8] as well as the crystal structures of TrmB [9] and TrmBL2 [10] show that both proteins share the same succession of homologous domains, a N-terminal DNA binding helix-turn-helix domain, a dimerization helix and a C-terminal domain. TrmB and TrmBL1 possess an additional domain at the C-terminus which is involved in binding of the effector sugar molecules.

While these structural findings suggest that TrmB and TrmBL2 have evolved from common ancestry, TrmBL2 still features a number of properties distinct from the other TrmB-like proteins. TrmBL2 is tetrameric when purified from *Pyrococcus furiosus* cells as well as in the crystal structures without DNA and with bound DNA. It possesses a highly conserved sequence [1] and is the only TrmB member that is found in all *Thermococcales*; a trend which has been confirmed by the growing number of genomes available. Other unique features of TrmBL2 are a constant and high expression level [1] with intracellular protein concentrations determined to be around 37 μM in *Thermococcus kodakarensis* [11] and the lack of the C-terminal sugar-binding subdomain present in TrmB and TrmBL1.

To this date the physiological role of TrmBL2 is unclear. The relation to the specific and global transcriptional family members TrmB, TrmBL1 and VNG1451C would suggest a regulatory role. Indeed, Maruyama et al. [12] reported binding of TK0471, the TrmBL2 orthologue in *T*. *Kodakarensis* (sharing 82% sequence identity), to coding and noncoding regions of the DNA. They determined derepression by up to a factor of 80 in a TK0471 deletion strain during the log phase for some promoter regions which bind TK0471. Atomic force microscopy revealed the formation of thick filaments on DNA of about 13 nm in diameter that displayed the same contour length as the bare DNA. Recently, Efremov et al. [11] used single-molecule mechanical experiments and showed that TK0471 binding stiffens double-stranded DNA (dsDNA) by a factor of 2.5. This suggests that the protein is a member of the chromatin organizing system. Its particular role remains, however, still elusive, since up to now no clear phenotype has been reported for the TK0471 knock-out strain [12]. A conceivable function for the protein would be the protection of DNA: At the optimal growth temperature for the members of the *Thermococcales* the DNA double helix is usually unstable [13]. Stabilization of the DNA against melting is therefore one possible role but other protective effects are conceivable.

Other known archaeal chromatin proteins are histones, which compact the DNA [14] and Alba (Acetylation Lowers Binding Affinity). The latter protein forms at high concentrations stiff filaments with dsDNA which retain the contour length of DNA [15] similarly as recently observed for TK0471 [11]. Alba association also shifts the DNA melting temperature towards higher values [16]. In addition it forms crossbridges between DNA and acts as a differential gene regulator [17]. Noteworthy is in this context, the *Escherichia coli* protein H-NS which also forms stiff filaments and cross bridges between DNA and acts as a transcriptional repressor [18,19]. However, neither for Alba nor for H-NS a clear link between its physiological functions and its physico-chemical properties has emerged.

To better understand the functions of archaeal chromatin proteins we investigated the binding of TrmBL2 to dsDNA using mechanical DNA manipulation by magnetic tweezers [20]. We demonstrate that TrmBL2 forms stiff filaments with dsDNA. Similar results have been oberved by Efremov et al. for the orthologue TK0471 from *Thermococcus kodakarensis* [11]. Measurements of the filament formation kinetics reveal the highly cooperative nature of this process. In contrast to Alba, cross-bridging between two DNA strands by TrmBL2 is not observed. To test a possible dsDNA stabilization by TrmBL2 we mechanically unzipped DNA hairpins in presence of the protein and found that TrmBL2 inhibited the disruption of the paired bases. Surprisingly, when bound to the ssDNA being produced during hairpin disruption, TrmBL2 inhibited double-helix formation similarly as single-strand binding proteins. Furthermore, the disruption experiments in combination with the recently determined atomic structure [10] provide evidence that the smallest broken protein-DNA interaction unit is a pair of DNA binding helix-turn-helix domains within the tetramer which bind to the same major groove. In summary, our tweezers data suggests that TrmBL2 stabilizes both dsDNA and ssDNA. Combining the structural data as well as the results of the mechanical experiments, we finally present a structural model for the TrmBL2-dsDNA filament.

## Materials and Methods

### Cloning, expression and purification of TrmBL2

TrmBL2 from Pyrococcus furiosus was cloned and expressed in *E*.*coli* BL21 (DE3) cells (New England Biolabs) which were cultivated in LB medium at 37°C as described previously [10]. Protein expression was induced by adding 1 mM IPTG to the cell culture at an OD600 of 0.6. The cells were grown for an additional 4 hours before harvesting. The cell pellet was stored at -80°C. For purification, 6g cells were resuspended in 40 ml lysis buffer containing 40mM HEPES at pH 7.5, 150 mM NaCl and 20% glycerol. Lysis was performed using a French press at 16,000 psi. The lysate was heated to 80°C for 25 minutes and centrifuged at 185,500 g for 60 minutes. The NaCl concentration of the supernatant was diluted to 50 mM before loading onto a Q-Sepharose anion-exchange column equilibrated with 40 mM HEPES pH 7.5 and 50 mM NaCl (Buffer A).The protein eluted between 180 mM and 200 mM NaCl upon applying a linear gradient of NaCl by mixing buffer A with buffer B (40 mM HEPES pH 7.5, 1 M NaCl). The protein containing fractions were identified by SDS-PAGE, pooled, concentrated and subjected to gel-filtration on a 60 ml Superdex 200 column equilibrated with 40 mM HEPES pH 7.5 and 150 mM NaCl. TrmBL2 eluted as a single peak corresponding to its dimeric form, in contrast to the tetramers obtained from *Pyrococcus furiosus* cells [10]. While this may be caused by differences in conditions in the bacterial and the archaeal cell, the crystal structure of dimeric TrmBL2 from *E*.*coli* cocrystallized with DNA shows a tetrameric structure which is almost indistinguishable from the TrmBL2 tetramer obtained from *Pyrococcus furiosus* and crystallized in absence of DNA [10].

### Preparation of DNA for Magnetic Tweezer Experiments

The dsDNA used for the Magnetic Tweezer experiments was prepared by digestion of a 11.3 kbp plasmid (pBluescript II SK+ into which DNA fragments from bacteriophage λ were cloned) using the restriction enzymes *Pci*I and *Sac*I, which provided a 10929 bp long linear DNA fragment. On one of its ends (*Sac*I overhang) a 600 bp digoxigenin-modified DNA handle and on the other end (*Pci*I overhang) a 600 bp biotin-modified DNA handle was ligated using T4 ligase to allow the binding of the DNA within an anti-digoxigenin modified flow cell and to a streptavidin coated bead (see below). Both handles were produced by PCR in presence of modified dNTPs and digested with the corresponding enzymes. The final product was purified by agarose gel electrophoresis.

To our knowledge none of the DNA constructs used in this work contain specific binding sites for TrmBL2.

The DNA hairpin used for the unzipping experiments consisted of a single biotin at the 5’-terminus, followed by a 11 bp ssDNA spacer, a hairpin with a 488 bp stem derived from λ-DNA, a 60 bp ssDNA spacer, a 1020 bp dsDNA spacer and a 632 bp dsDNA containing multiple digoxigenin labels. It was prepared as previously described [21].

### Magnetic Tweezers experiments

Magnetic tweezers experiments were carried out in a home-built magnetic tweezers apparatus as described in detail before [22]. Briefly, our setup contained two rare-earth magnets (NdFeB magnets W-05-N50-G; Supermagnete, Uster, Switzerland) aligned through a motorized platformed above the sample stage of a home-built inverted microscope. The three-dimensional positions of the magnetic beads and non-magnetic reference particles were determined in real-time at 300 Hz using videomicroscopy and fast image processing [23]. Forces were calibrated using the “long-pendulum” geometry as recently described [24]. Measurements were performed in a custom-built flow cell of approximately 50μl volume consisting of 2 glass coverslips separated by a layer of parafilm which had the measuring chamber cut out.

The flow cell was functionalized using 30 μl anti-digoxigenin solution (c = 50 μg/ml, Roche) by incubation for 1h at room temperature. Passivation was performed by adding 60 μl BSA solution (c = 10 mg/ml, NEB) and incubation over night at 4°C. Streptavidin-coated magnetic beads (Dynabeads® MyOne T1 or Dynabeads M280) were incubated with the DNA tether for approximately 10 minutes at room temperature and flushed into the flow cell to allow binding of the digoxigenin-modified DNA end. After removal of unbound beads magnetic tweezers measurements were started. All experiments were performed in phosphate-buffered saline (PBS) at pH 7.4 supplemented with 5 mM NaN_3_.

Correct attachment of the tether to the magnetic bead via a single intact dsDNA molecule was verified by the asymmetric shape of the rotation curve at 1 pN.

Correct attachment of the DNA hairpin was confirmed by measuring the unzipping force of the hairpin and lengths of the opened and closed form of the hairpin at several forces.

Removal of the protein between measurements with different TrmBL2 concentrations was ensured by flushing the flow-cell with 1 M NaCl and 20 volumes PBS afterwards. A force-extension curve was recorded prior to each measurement to ensure complete removal of the protein.

### Electrophoretic Gel Shift Assay (EMSA)

For gel shift assays, a 60 nt oligonucleotide (5´-GATATAATATACCTATATCAATGGCCTCCCACGCATAAGCGCAGATACGTTCTGAGGGAA) labelled with Cyanine 5 (Cy5) at the 5’-end containing no TGM DNA binding motif for TrmBL2 [25] was annealed with a complementary strand (10 μM end concentration) in 1x Tris-borate-EDTA buffer (TBE), 10 mM MgCl_2_ and 100 mM NaCl by heating to 100°C and slow cooling in a thermobox to room temperature. The annealing was confirmed by running the resulting product on an 8% polyacrylamide gel (PAGE) in 0.5x TBE buffer.

Binding reactions with TrmBL2 were performed in PBS with a final concentration of 10 nM DNA and the respective protein concentration. Reaction solutions were incubated for 1 h at room temperature and protected from light before loading onto a polyacrylamide gel (8% 19:1 acrylamide-bisacrylamide, PBS). Gels were imaged using the Cy5 fluorescence signal for the labelled DNA and stained with Ethidium bromide (EtBr). Each lane was loaded with 20 μl DNA solution.

### Data analysis

Force-versus-extension data were modeled as previously described [26,27] assuming an inextensible semi-flexible polymer (worm-like-chain, WLC). Fitting the experimental data yielded two parameters: the persistence length and the contour length of the attached DNA/filament.

In single-molecule and EMSA measurements we quantified the fraction *f* of DNA bound by the protein, referred to as ‘coverage’ hereafter, as function of the TrmBL2 concentration [15] and fitted the data with a Hill equation according to

$$f=\frac{1}{1+{\left(K_{D}/c\right)}^{p}}$$

where *c* represents the protein concentration, *K*_D_ the dissociation constant and *p* the factor of cooperativity.

## Results

### Formation of a stiff filament of TrmBL2 bound to dsDNA

**Fig 1:**
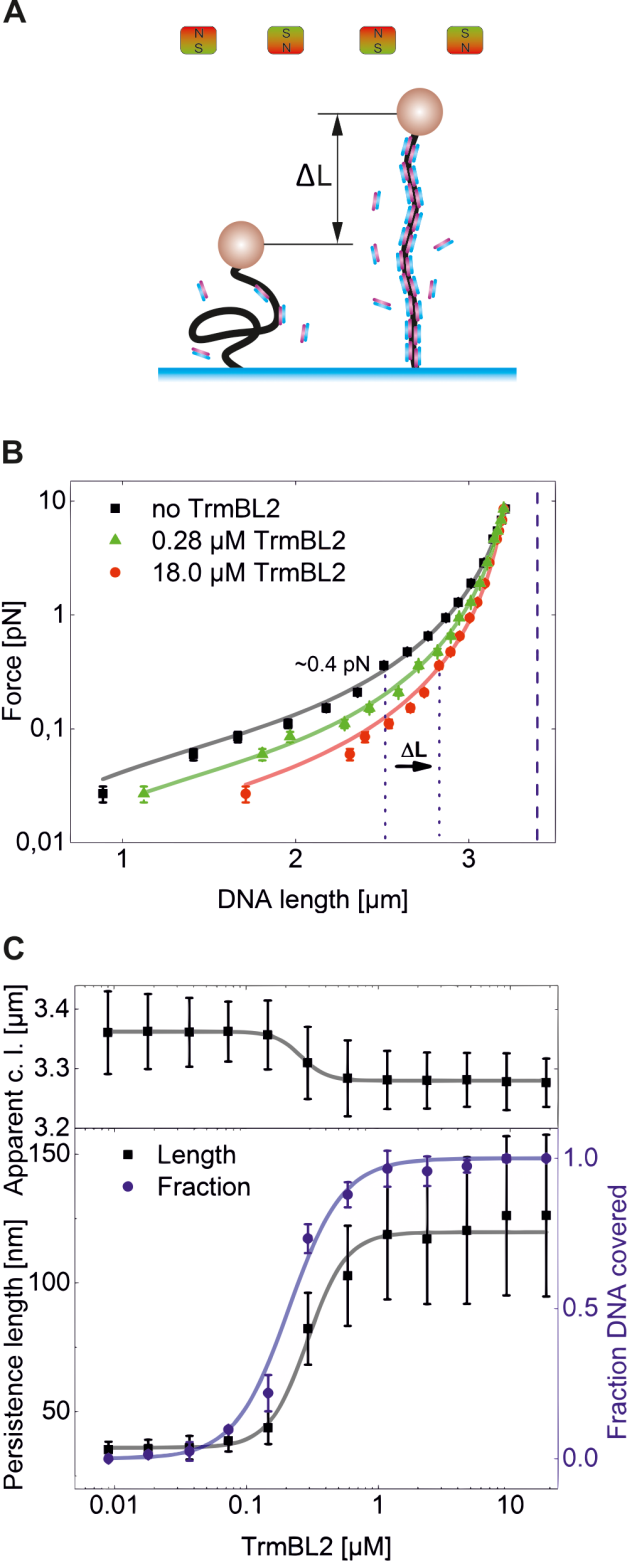
TrmBL2-filament formation on dsDNA. **(A)** Schematic representation of dsDNA length change as result of TrmBL2 binding. In the absence of protein (left) the bare DNA is in a random coil configuration at low persistence length. Upon protein binding the stiffness of the DNA increases markedly, resulting at low forces in a more extended DNA conformation seen as an increase in the end-to-end distance by ΔL (right). **(B)** Force extension curves of dsDNA in the absence of TrmBL2 (black squares) and presence of 0.28 μM (green triangles) and 18 μM (red circles) TrmBL2 for a single bead. Fits of the data with the WLC model (shown as solid lines) provided, respectively, filament contour lengths of 3.36 ± 0.07 μm, 3.31 ± 0.06 μm and 3.28 ± 0.04 μm (blue dashed line) as well as persistence lengths of 36.4 ± 2.9 nm, 82.17 ± 14.0 nm and 126.13 ± 31.4 nm. ΔL indicates the length change expected at a force of 0.4 pN as applied in the kinetic measurements (Fig 2). **(C)** Filament contour length (top panel), persistence length (black squares, bottom panel) and DNA coverage (blue circles, bottom panel) as function of the TrmBL2 concentration. The coverage was calculated using the persistence lengths obtained from force extension data. A fit to the data from four beads (solid grey and blue lines) provided a half-coverage concentration of 0.3 ± 0.2 μM and a Hill coefficient of 3.0 ± 0.33 for the DNA coverage. The error bars resulted from averaging apparent contour length, persistence length and coverage from four different beads.

To gain insight into the TrmBL2-DNA interaction we characterized the binding process using magnetic tweezer experiments. In particular, we carried out force-extension measurements, which monitor the applied force as function of the DNA end-to-end distance. To this end we anchored 10,929 bp long DNA molecules at one end to the bottom glass surface of a flow cell and at the other end to superparamagnetic beads (Fig 1A). A pair of magnets above the surface allowed to exert force onto the beads. The end-to-end distance was determined from the bead positions in axial direction that were obtained in real-time using high resolution videomicroscopy [23,28]. Forces were calibrated using the lateral fluctuations of the beads using the so-called “long-pendulum” geometry [24].

In absence of force long DNA molecules in solution adopt a random coil conformation in which free energy contributions from entropy and DNA bending are balanced. Applying external forces favour extended DNA conformations. A fit based on the worm-like-chain model [26,27] of the characteristic force-versus-extension behaviour provides the persistence length as well as the contour length of the attached polymer tether. The persistence length is directly proportional to the bending rigidity of the DNA and can be considered as the length over which the polymer still behaves as a straight rod. Upon binding of TrmBL2 both the persistence length and the contour length may change and differ from that of bare DNA.

First we measured the force-extension behaviour of the dsDNA [29] in absence of TrmBL2 in PBS buffer at pH 7.4 (Fig 1B). For the bare dsDNA tether a persistence length of 36 ± 2.9 nm and a contour length of 3.36 ± 0.07 μm were found. When adding TrmBL2, we took care to start measurements after the binding reaction had reached equilibrium. Upon protein addition the persistence length started to increase at a concentration of 9 nM until saturation was reached at 1 μM TrmBL2 concentration. At 18 μM TrmBL2 a persistence length of 126 ± 31.4 nm was obtained (Fig 1C). The increase of the persistence length, i.e. bending rigidity, in presence of TrmBL2 is indicative of the formation of a DNA-TrmBL2-filament. The contour length from the WLC fit decreased slightly by approximately 2% (70 nm) (see Fig 1C top) over the whole concentration range. An almost unchanged contour length is in agreement with atomic force microscopy (AFM) data for the TrmBL2 orthologue TK0471 [12], taking the limited accuracy of these data into account. While TrmBL2 binding slightly decreased the contour length at high forces, a pronounced DNA length increase was observed at low forces (< 0.5 pN) due to the stiffening of the DNA.

From the measured persistence length change we calculated the protein coverage on the DNA (Fig 1C) as function of the protein concentration [15]. Fitting these data with a Hill equation resulted in a half-coverage concentration of 0.3 ± 0.01 μM and a Hill coefficient of 3.0 ± 0.3. Similar data but obtained for TK0471 was previously fitted with a double Hill equation to account for a minor coverage increase close to saturation [11] indicative of a second binding mode. For our data such a coverage increase may exist as well between 5 and 10 μM. However, this increase was within the measurement errors and involved only few data points, such that reliable fitting was precluded. Force-extension curves allow to detect protein-induced cross-bridges between two dsDNA strands, which are seen as numerous sudden disruption events at distances below the contour length of the tether. These sudden disruptions are due to the breakage of protein-induced loops as observed e.g. for Alba at concentrations of 1–100 nM [15]. We did not observe disruption events in any of our experiments, indicating the absence of cross-bridging capability of TrmBL2 in agreement with results obtained for TK0471 [11].

### Kinetics of TrmBL2 binding to dsDNA

**Fig 2:**
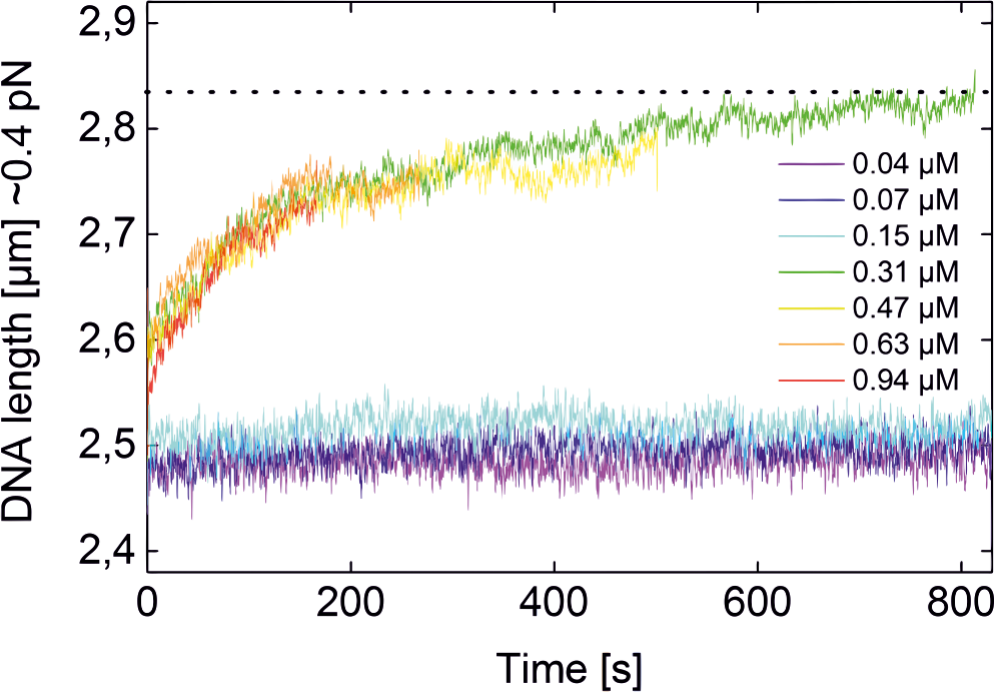
Kinetics of TrmBL2 binding to dsDNA at different concentrations of TrmBL2. The kinetics were measured by monitoring the change of the DNA length at a force of 0.4 pN, which linearly changes with the coverage of the DNA by the protein. The data shown were recorded from the same DNA molecule. At room temperature, the filament formation is completed (95%) within approximately 10 minutes and the final DNA length (dotted line) is observed.

One explanation for the gradually increasing coverage of the DNA with increasing protein concentration is that the filament is in rapid equilibrium with TrmBL2 in solution. To test this possibility we measured the kinetics of TrmBL2 filament formation. To this end we monitored the DNA length at 0.4 pN, which due to the stiffening of the DNA tether will increase upon filament formation (see above). The binding kinetics of the protein to dsDNA measured at different protein concentrations (Fig 2) were in agreement with a significant cooperativity of the process: For concentrations below 0.3 μM no filament formation was observed within 800 s while at higher concentrations pronounced filament formation occurred at a rate that was largely independent of the protein concentration. Overall TrmBL2 filament formation was a relatively slow process (95% of the extension change was accomplished in only 10 minutes, Fig 2).

After removing TrmBL2 from solution by flushing the flow cell extensively with buffer no filament dissociation was observed even over a period of 12 h. This shows that the TrmBL2 binding to DNA is mostly kinetically determined. Partially covered DNA (Fig 1C) is thus the result of individual stable TrmBL2 filament patches.

### Supercoiling of TrmBL2-DNA filaments

**Fig 3:**
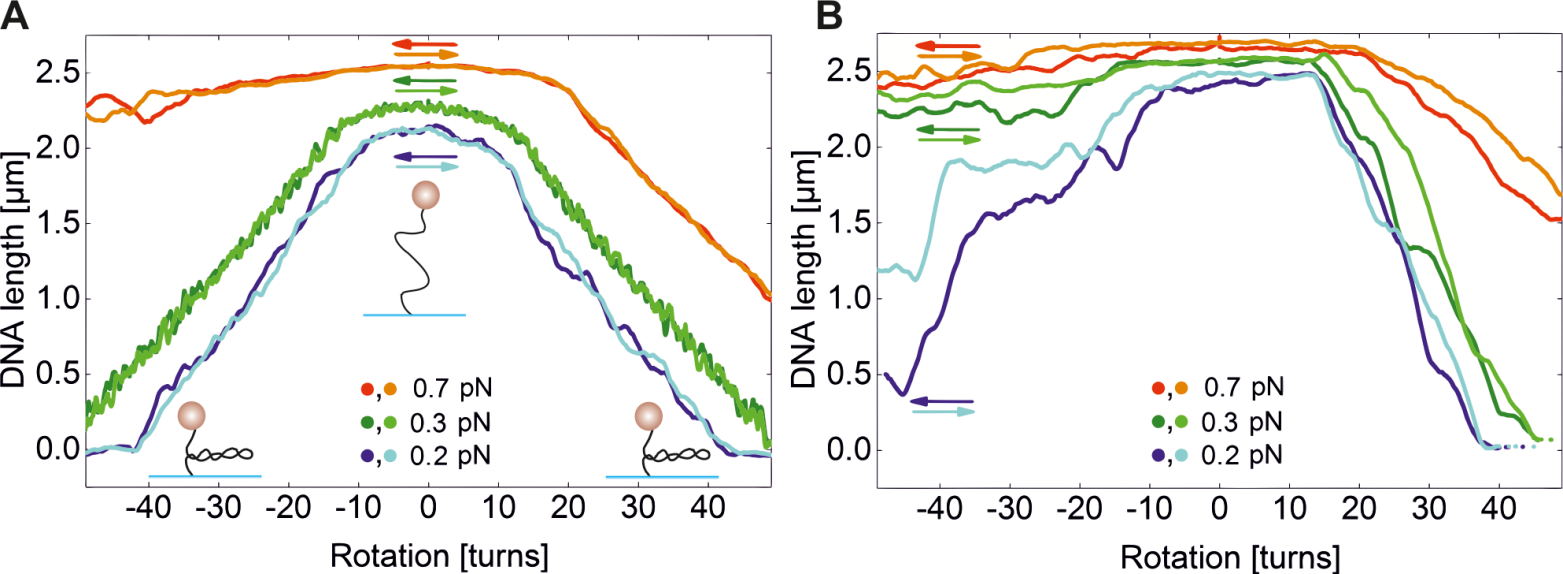
Altered supercoiling response of dsDNA in presence of TrmBL2 for different forces. Arrows indicate the sense of rotation whereas the colour indicates the applied force. **(A)** Supercoiling curves in absence of TrmBL2 at different forces. The lighter and darker shade of a given color show curves that were obtained when inducing negative and positive supercoiling, respectively. Small cartoons of the plectoneme formation are shown. **(B)** Supercoiling curves taken at the same conditions in presence of 4.6 μM TrmBL2. The presented curves were all recorded using the same tether.

*P*. *furiosus* possesses a reverse gyrase suggesting positive supercoiling of the DNA [30]. Supercoiling plays an important role during most DNA transactions, among them replication and transcription. We therefore investigated how TrmBL2 filament formation affects DNA supercoiling. To this end we twisted single DNA molecules held at constant force by rotating the magnets around the z-axis. For bare DNA, the length changed upon twisting in a characteristic, previously described manner [31–33]: Initially, the molecule length remained constant while the torque within the molecule increased linearly. Once a critical torque was reached, the molecule buckled and a plectonemic superhelix formed such that the length decreased linearly with the applied turns (Fig 3A), while the torque remained constant [34]. With increasing force, the twist at the buckling point as well as the torque in the plectonemic phase were also increased. At forces of 0.5 pN and below the obtained supercoiling curves were symmetric with respect to positive and negative supercoiling. At higher forces (up to 5 pN) plectoneme formation still occured for positive supercoiling but was impeded for negative supercoiling (Fig 3A). At these forces the critical torque for DNA untwisting was reached, such that negative supercoiling is absorbed by structural transitions such as the formation of denaturation bubbles or Z-DNA.

When carrying out supercoiling experiments in presence of 4.7 μM TrmBL2 (where full DNA coverage is expected), an overall similar supercoiling response of the protein-DNA filament compared to bare DNA was observed (Fig 3). In particular, the maximal length that corresponds to a torsionally relaxed filament was not shifted along the turn axis. This indicates that TrmBL2 does not un- or overtwist DNA in agreement with the atomic structure [10]. There are however also noticeable differences between the supercoiling response of the filament and bare DNA: (i) Already at forces of 0.2 and 0.3 pN the curves became asymmetric, most likely due to structural transition within the DNA at negative supercoiling. (ii) The slopes of the supercoiling curves appeared steeper. (iii) The buckling point was shifted slightly towards higher turns for positive supercoiling at 0.2 pN (from 12 to 13 turns) and 0.3 pN (from 14 to 15 turns). On first glance, the asymmetry of the supercoiling curves at low forces would suggest that TrmBL2 destabilizes DNA. However, in the twisting experiments only a known superhelical density was applied that depending on the torsional stiffness of the attached molecule can translate into very different torque values. We previously developed a theoretical model that allows to estimate the torque in the plectonemic phase based on the persistence length and the radius (including electrostatic repulsion layer) of the semiflexible chain as well as the applied force [35]. For bare DNA a torque in the plectonemic phase of 9.3 pN nm was calculated for 0.7 pN, at which structural DNA alteration occured at negative supercoiling. For the TrmBL2 filament with a persistence length of 120 nm and a filament radius of 10 nm (see structural model below) such a torque will already be reached at 0.25 pN. For bare DNA a torque of only 5.3 pN nm is obtained at 0.25 pN. This indicates that the torsional stiffness of the TrmBL2 filament is significantly larger than that of dsDNA since the buckling point is not much changed upon protein binding. Overall, the significantly increased stiffness of the TrmBL2 filament with respect to torsion and bending are responsible for the observed structural transitions at low force, since they cause higher torque values at small superhelical densities. *In vivo* however, DNA regions covered by TrmBL2 are most likely connected in series to TrmBL2 free regions, that are less torsionally rigid. In this configuration the same torque acts on each region, such that most of the induced turns are absorbed by the soft regions rather than by the TrmBL2 filament. In this scenario TrmBL2 is therefore preventing plectoneme formation as well as supercoiling of the DNA within the filament.

### Mechanical Stabilization of DNA by TrmBL2

**Fig 4:**
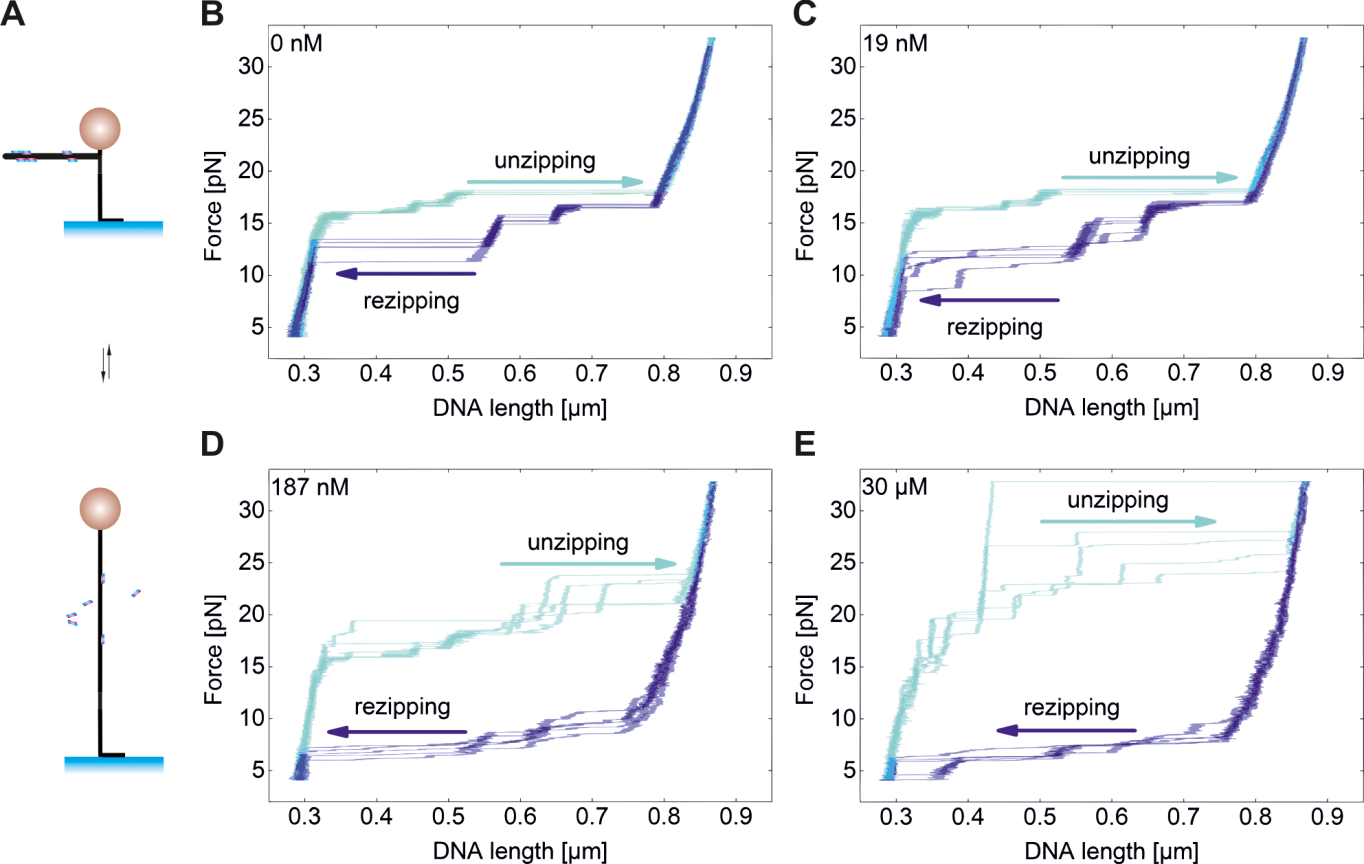
DNA unzipping in absence and presence of TrmBL2. A DNA hairpin is anchored through a dsDNA spacer at one end to the surface and at the other end to the magnetic bead (top). At sufficient force the hairpin is disrupted. When lowering the force, the hairpin spontaneously closes. **(B-E)** Unzipping (cyan) and rezipping (blue) cycles of the hairpin (see arrows for direction) in absence and presence of TrmBL2 as indicated in the figures. Hairpin rezipping is already affected at low TrmBL2 concentrations (19 nM) while hairpin unzipping is affected only when significant fractions of the DNA are covered by the protein (from 187 nM on). This shows that bound TrmBL2 shifts the unzipping to higher force and the rezipping to lower force indicating that the protein binds both, dsDNA and ssDNA.

To investigate whether TrmBL2 stabilizes dsDNA against denaturation, we performed magnetic tweezers experiments using a 488 bp long DNA hairpin. In particular, we carried out cycles of hairpin unzipping-rezipping experiments in absence of protein, in which the force was slowly increased until unzipping occurred and subsequently slowly decreased to monitor hairpin refolding (Fig 4B). The resulting force-versus-extension trajectories exhibited a pronounced hysteresis (Fig 4B), while the hairpin was completely unzipped at 18 pN complete rezipping was only observed between 13 to 11 pN. The reproducibility of this behaviour was confirmed in repetitive cycles of the experiment (Fig 4B). Slight differences were only seen for individual partial unzipping/rezipping events in which a longer hairpin section abruptly (un-)zips, due to the stochastic nature of these processes.

We next repeated the experiment in presence of 19 nM, 187 nM and 30 μM TrmBL2, conditions that, respectively, corresponding to less than 1%, 44% and full coverage (>99%), respectively, of the DNA by the protein (see Fig 1C). At 19 nM TrmBL2 the unzipping of the hairpin was not affected, while the closing of the hairpin was shifted to lower forces in some cases (Fig 4C). At concentrations of 187 nM and 30μM, hairpin closure was significantly impeded and only occurred at reduced force of 5–6 pN (Fig 4D and 4E). Furthermore, the hairpin opening forces were also found to be increased, most pronounced for the fully covered DNA at which forces between 23 pN and 32 pN were required for full unzipping.

Overall the hairpin experiments suggest that TrmBL2 binds and stabilizes dsDNA as well as ssDNA.

**Fig 5:**
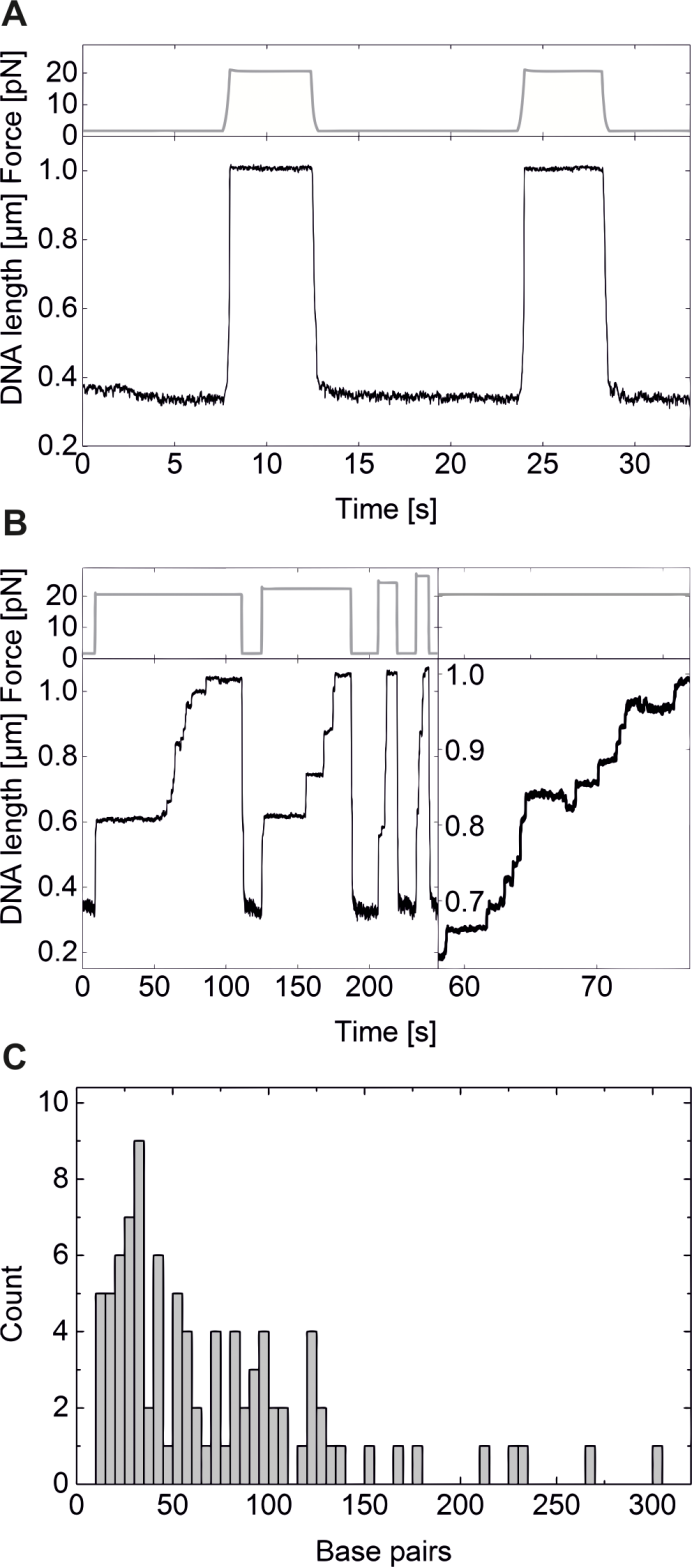
Hairpin unzipping at constant force reveals discrete protein dissociation events. **(A)** Time trajectory of DNA hairpin unzipping and rezipping in absence of TrmBL2. The force was alternated between 20.6 pN and 1.6 pN to induce the conformational changes of the hairpin. Hairpin unzipping occurred under these conditions in a single fast step. **(B)** Time trajectory of DNA hairpin unzipping and rezipping in presence of 30 μM TrmBL2. Different forces from 20.6 pN to 26.5 pN were used for unzipping (see force axis). Hairpin opening occurred in small steps obviously due to displacement of bound protein (see detail in the right panel). **(C)** Number of base pairs unzipped in single steps for the traces shown under (B). The heights of the steps were determined manually and converted into the number of opened base pairs with a force-dependent algorithm (unpublished).

During unzipping of fully covered DNA, many discrete steps were visible (Figs 4E and 5B) that were not observed in absence or low concentrations of TrmBL2 (Figs 4B, 4C and 5A). To reveal how TrmBL2 dissociates from dsDNA, we carried out unzipping experiments at constant force (Fig 5B). We then analyzed the size of the small abrupt steps that occurred during hairpin opening. Only steps between states of constant tether length which lasted for more than 0.5 seconds were evaluated. The step sizes were converted to the number of unzipped base pairs and compiled in a histogram (Fig 5C). Step sizes of less than 10 base pairs were not observed. It can therefore be concluded that in these cases 10 subsequent base pairs are breaking virtually simultaneously (Fig 5C). Ten base pairs correspond to the distance between two successive major grooves. In the crystal structure of tetrameric TrmBL2 bound to dsDNA one pair of ewHTH domains binds to a major groove and the other pair to the adjacent major groove. This suggests that the smallest dsDNA unzippings occur when one pair of ewHTH domains dissociates from DNA while the other pair maintains DNA association. The majority of the steps, however, occurred at sizes of 20–100 bp, suggesting that the dissociation of a TrmBL2 tetramer or higher oligomers is preferred over dissociation of only one pair of ewHTH domains in one tetramer.

### Affinity to dsDNA and ssDNA

**Fig 6:**
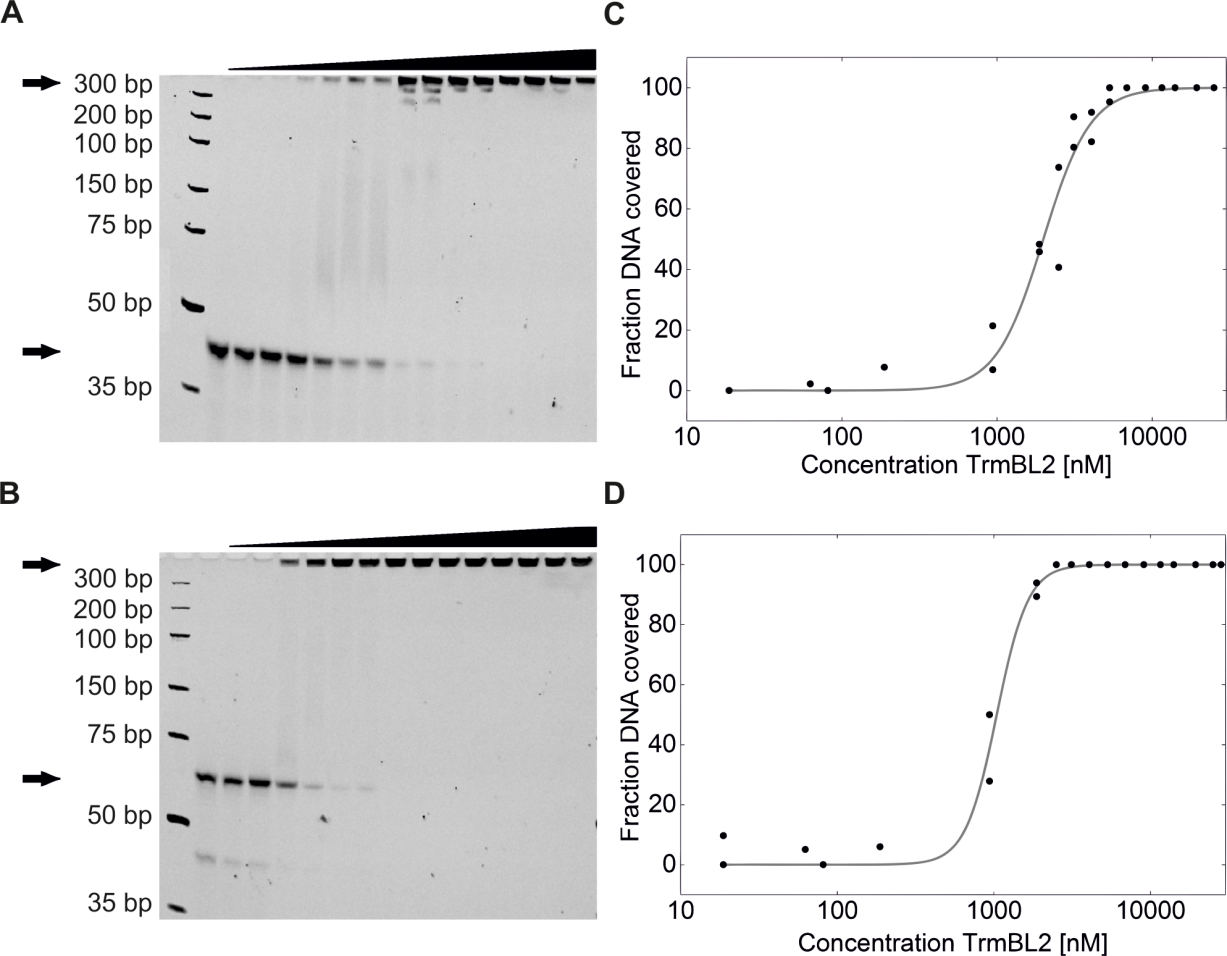
Gel shift assay of TrmBL2 binding to ssDNA and dsDNA. The lanes contained TrmBL2 at increasing concentration (solid black ramp) and DNA. **(A)** Gel of ssDNA incubated with TrmBL2 at different concentrations. Marked with arrows are the ssDNA/TrmBL2 complex (top) and the ssDNA alone (bottom). **(B)** Gel of dsDNA incubated with TrmBL2 at different concentrations. Marked with arrows are the dsDNA/TrmBL2 complex (top) and the ssDNA alone (bottom). Note that unhybridized ssDNA runs at lower molecular weight. The bare dsDNA sample shows 2 bands, one annealed dsDNA band and one ssDNA band which resulted from unhybridised residual 60mer. The unhybridised band made up approximately 5% of the total fluorescence. **(C)** Titration curve using measured intensities of the bands indicate a K_D_ of 2.0 μM for ssDNA. Data were fitted using a logistic binding model. **(D)** Titration curve after analysis of band intensities for TrmBL2 binding to dsDNA indicate a K_D_ of 1.0 μM. Data were fitted using a Hill equation.

To further elucidate binding of TrmBL2 to ssDNA and dsDNA and to determine apparent binding affinities, we performed electrophoretic mobility shift assays (EMSA). To this end we used a synthetic 60mer DNA oligomer (see methods) labelled on its 5’-ends with Cy5. A secondary structure prediction for this oligonucleotide did not reveal any stable secondary structure at the experimental conditions applied. For the dsDNA measurements this oligomer was additionally annealed to a strand with complementary sequence. After incubation of ssDNA and dsDNA with a sufficient concentration of TrmBL2 for 1 h a slower migrating DNA species appeared for both DNA species that was attributed to the TrmBL2-DNA complex (Fig 6A and 6B). We quantified the fraction of protein-bound DNA as function of the TrmBL2 concentration, the coverage, and fitted the data with a Hill equation (Fig 6C and 6D; see Methods). This provided apparent *K*_d_ values of 2.1 ± 0.1 μM and 1.07 ± 0.03 μM as well as Hill coefficients of 2.9 ± 0.4 and 4.4 ± 0.6 for the binding to ssDNA and dsDNA, respectively. These results are in good agreement with the apparent *K*_d_ for dsDNA binding from the mechanical measurements given the different lengths of the DNA substrates and the cooperativity of the binding process. Also the affinity of TrmBL2 to ssDNA that is comparable to dsDNA binding, which corroborates well the observed impeded closure of hairpin DNA. The rather high affinity to ssDNA is quite surprising but not uncommon [36]. ssDNA binding proteins generally function in DNA replication [37] and repair [38]. This suggests that TrmBL2 could also stabilize ssDNA in cooperation with dedicated ssDNA proteins but no respective report is known to us.

### Modelling the filament from the TrmBL2 crystal structure

The magnetic tweezers measurements presented here for TrmBL2 as well as of its orthologue TK0471 [11] indicate the formation of a stiff filament. This implies, that these proteins stabilize an extended conformation of the DNA helix and do not introduce significant overall curvature.

**Fig 7:**
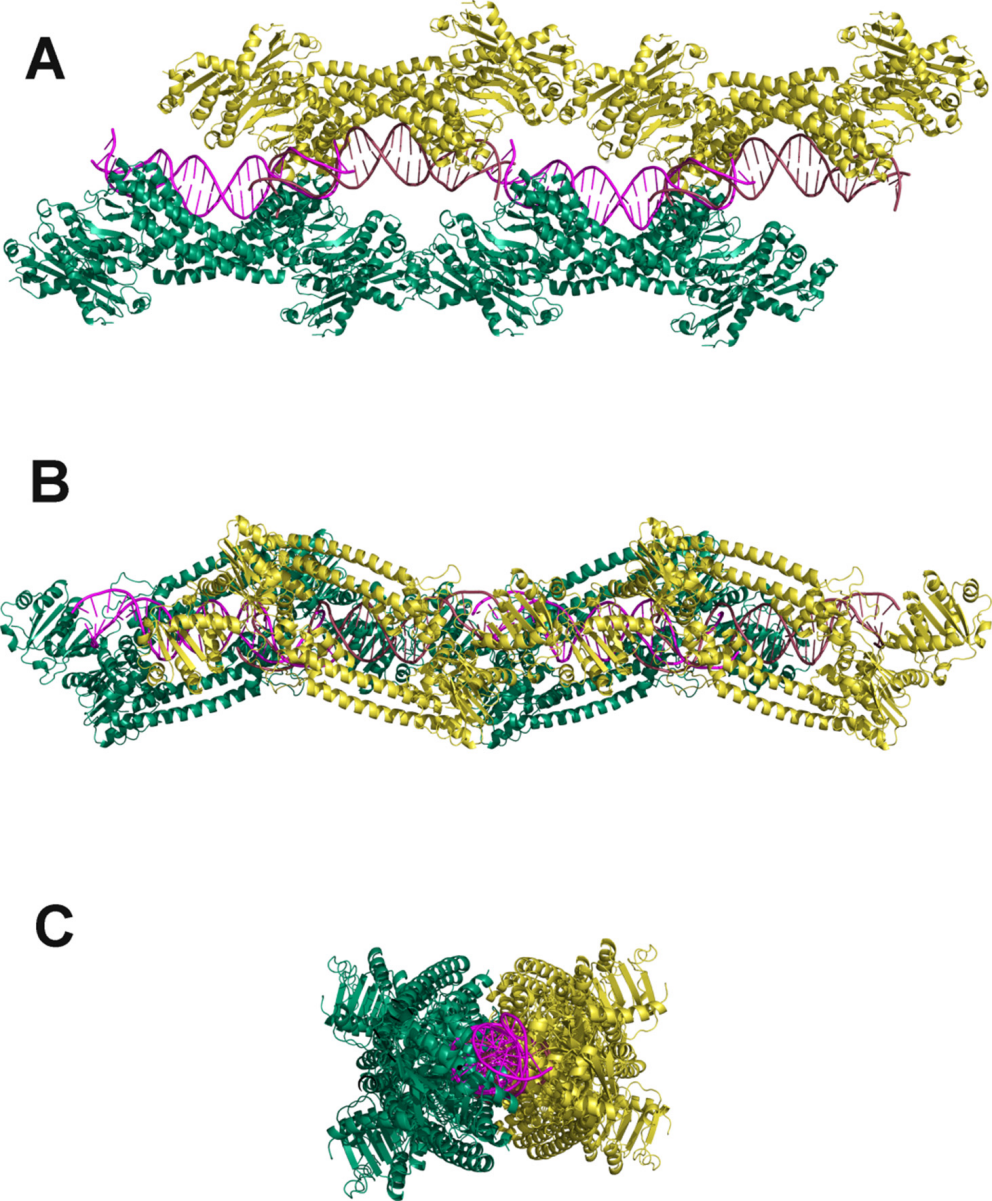
A model for the TrmBL2 filament using the crystal structure of TrmBL2 with bound dsDNA. In the crystal structure of TrmBL2 from *Pyrococcus furiosus* [10] four protein molecules bind to the 19 basepairs long TGM dsDNA. In the filament model shown in panel A, it comprises the first quarter with DNA (pink) and the protein tetramer (green). First an octameric complex was constructed from two copies of the crystal structure. In the uppermost panel it comprises the left halve with one pink, one red DNA piece, one green and one yellow tetramer. Two copies of the octameric complex were shifted in tandem to get a continuous dsDNA running through both as seen in panel A. The resulting dsDNA in the hexadecamer has zero overall curvature. The hexadecameric filament model is shown in ribbon representation in three orthogonal views (A-C). Note the almost full coverage of the DNA by TrmBL2 and that the DNA is in an almost perfectly linear conformation.

Using this information we developed a model for the TrmBL2-DNA filament based on the crystal structure of the tetrameric TrmBL2 in complex with DNA. The structure of TrmBL2 [10] shows that the ewHTH domain at the N-terminus is followed by a long amphipathic helix which promotes dimer formation and places the ewHTH domains suitably to interact with two adjacent major grooves of dsDNA. The dimers further oligomerize to form a tetramer which was co-crystallized with the 19 base pair long dsDNA *T**hermococcales* glycolytic motif (TGM). The electron density showed 25 base pairs which could not be unambiguously identified. The refinement succeeded by assuming that 3 copies of the DNA are observed as an ensemble average in the crystal structure. This would be in agreement with the ability of TrmBL2 to bind non-specifically to DNA at elevated concentrations. Furthermore, the structure shows that the protein tetramer imposes a bend on these approximately 2 turns of DNA amounting to 63° [10]. In order to construct a model for an elongated filament which compensates for this bending, we joined one copy of the tetramer-DNA complex to a second one which was rotated by 180° degrees around the helical axis in the center of the complex. We positioned the two complexes such that the local helix axes at the junctions matched each other and the two DNA helices overlapped by half a helical turn. In this octameric model the two tetramers show few steric clashes (Fig 7A). The DNA in the resulting TrmBL2 octamer is forming approximately 3.5 helical turns and possesses no overall curvature, even though the DNA path undulates slightly.

The octameric structure was taken as repetitive unit of the filament, i.e a copy of it was shifted so that the DNA should run continuously through the resulting TrmBL2 hexadecamer and that steric clashes of the protein are kept to a minimum. This tandem formation was achieved with little gaps between the individual DNA molecules (Fig 7A and 7B). Assuming a sinusoidal undulation of the DNA the model predicts a contour length for the filament of 97% of the DNA contour length which is in agreement with the observed protein dependent change of the filament length (see Fig 1C top). The filament thickness varies between 7 and 11 nm. The model is also in agreement with the observed minimal unzipping of 10 basepairs. We would like to point out, that our model does not represent an atomic resolution structure and has to be validated by future experiment. However, we believe that the overall architecture of the filament will be described by the model. The existing restrictions from experimental data, i.e. the crystal structure, the formation of a straight, stiff TrmBL2 filament with a contour length that is 2% shorter than for dsDNA and the minimal unzipping of 10 basepairs leaves only little room for other arrangements. Slightly different structures are nonetheless possible. For instance, there could be a rotational offset between adjacent octameric units in order to optimize protein-protein contacts, which would result in a helical arrangement of the octamers. Moreover, conformational changes due to induced fit upon contact formation between adjacent tetramers and octamers could modify the structure without causing gross deviations from the model. Nonetheless, all possible models that are conceivable with the octameric unit result in a stiff and on average straight filament in which the proteins cover almost the entire DNA surface (see Fig 7C). The solvent accessible surface of DNA in this model is reduced by only 20% compared to bare DNA [10]. The tetrameric repressor TtgV which binds specifically with its four wHTH domains to a longer DNA operator sequence of 42 bp [39] achieves the same relative reduction of the solvent accessible area showing that TrmBL2 contacts the DNA less intimately in absolute terms.

## Discussion

Using single-molecule mechanical measurements we showed here that TrmBL2 forms a rigid filament with dsDNA that exhibits increased rigidity against bending and twisting of dsDNA. Furthermore, TrmBL2 does not form crossbridges between distal DNA sites, which is different to Alba. Similar findings were reported by Efremov et al. for the orthologous protein TK0471 from *T*. *kodakarensis* [11]. Most likely TrmBL2-DNA filaments also occur *in vivo*, since such structures have been found for TK0471 in AFM investigations of chromosome fractions of broken cells [12]. Moreover, TK0471 is strongly expressed at a cytoplasmic concentration of 37 μM [11], which is much above the thresholds observed in this study for yielding full DNA coverage by the protein. The high and constitutive expression of TK0471 suggests that TrmBL2-DNA filaments play an important physiological role.

While Efremov et al. further showed impairment of TK0471 binding to DNA caused by the histone protein HTkB and by potassium, our experiments revealed further effects of TrmBL2 binding on DNA. Most surprisingly we found that TrmBL2 binds dsDNA and ssDNA with similar affinity and stabilizes ssDNA against DNA duplex formation. For the EMSA experiments an oligonucleotide with negligible secondary structure was used. Furthermore, ssDNA secondary structures are suppressed when applying forces between 5–10 pN [40]. Since the first inhibition events of hairpin rezipping appear at forces >10 pN, the observed binding in these experiments should be specific to single-stranded DNA rather than occur on secondary structures (e.g. small hairpins). Therefore, we think that TrmBL2 can assist other ssDNA binding proteins like H-NS [36] to protect single-stranded nucleic acids. This potential new function requires, however, further support in the future.

Furthermore we found that TrmBL2 stabilizes dsDNA against the disruption of base pairing. Simultaneously with saturation of the dsDNA binding sites with TrmBL2 the contour length of the filament decreased by approximately 2% compared to DNA alone. This suggests that the crystal structure of tetrameric TrmBL2 with bent DNA [10] matches part of the stiff and straight filament structure and that consecutive tetramers in the filament induce opposite bends. Using this we propose a structural model of the filament which must be close to the true structure. The model predicts that the protein sheath still leaves the DNA accessible for small molecules like water but that it is shielded from larger molecules like proteins. In agreement with this, the chromatin fraction in a TK0471 deletion strain where DNA shielding would be abrogated showed an increased MNAse sensitivity [12].

In our unzipping experiments the disruption of TrmBL2-DNA filaments occurred in a step-wise manner. The data suggest that the smallest interaction unit of TrmBL2 with DNA which is disrupted is an adjacent pair of ewHTH domains from one tetramer. The data also suggest that TrmBL2 often dissociates as a larger oligomer.

The embedding of the dsDNA by the protein can explain the observed stability of the double helix in the filament against dissociation into single strands. Moreover, the reduced thermal movements of DNA within this “protein sheath” could protect covalent bonds in the DNA against transient deformations which cause strain and as a result may lead to bond breakage or vulnerability towards nearby reactive groups [41]. Filament formation by TrmBL2 association thus could protect DNA in a similar way as vitrification in cryopreservation protects cells [42]. At low concentrations TrmBL2 covers only higher affinity sites on the DNA which can explain the repression of some promoters [12].

In spite of different basic structures, Alba proteins in the crenarchaeal phylum [17,43] and the histone protein H-NS found in bacteria, share with TrmBL2 the ability to form a stiff filament on DNA. Alba binding has been characterized as non-specific, but for an Alba homolog a binding sequence has been identified [44]. H-NS binds to short AT-rich sequences [45,46]. A mild derepression of certain genes upon knockout has been reported for H-NS as well [45]. It is therefore conceivable that they all have evolved from ancestors which control transcription at lower protein concentrations. For TrmBL2 this relationship is most clear from its kinship with the other TrmB family proteins which serve as specific repressors. TrmB binds to the MD operator at around 1 μM [2]. In the TK0471 deletion strain KCP of *T*. *kodakarensis* a few ORF transcript signals were upregulated by factors up to 80 [12], i.e. in the order of magnitude known for typical repressors [47].

The ability to form filaments with DNA obviously requires unspecific DNA binding. This unspecific mode of binding may well be shared by specific repressor molecules. It has been shown that the paradigmatic LacI repressor in absence of the inducer attaches with high-affinity to its specific operator binding site but in presence of the inducer it exhibits low- and nonspecific affinity to DNA allowing diffusion along the double helix [48,49].

Histones that effectively condense DNA and Alba1/Alba2 dimers that either crossbridges DNA or form filaments are thought to contribute to the structure of the genome. The dual binding modes of Alba have furthermore been suggested to play a regulatory function in modulating gene accessibility. For TrmBL2 it remains less clear, whether it contributes to the overall genome architecture, since it lacks a clear DNA condensation mechanism. Nonetheless, stiff filaments within a genome will certainly impact on the structure of the genome. Potentially, segments of stiff filaments are interspersed by softer “kinkable” regions that are more easily to compact, since such a configuration displays less degrees of freedom compared to a fully flexible chain.

In summary we propose that TrmBL2, Alba family proteins and H-NS family proteins have protective functions by forming stiff filaments with DNA due to low-affinity unspecific binding and, furthermore, unspecific binding and transcriptional control by specific binding seem to be interconvertible in evolution. The stiff coating may stabilize the DNA against a variety of possible damages. Specifically, our results with TrmBL2 suggest that it might prevent the disruption of the DNA double helix, e.g. avoid heat induced melting at the high growth temperatures of 100°C which are typical for the host organisms. Furthermore, the TrmBL2 sheath may shield DNA from contact by other proteins

Further work has to show to what extend such filaments contribute to survival under hostile growth conditions, gene regulation and genome shaping.
